# Supplementary figures and images for: How conformity can lead to polarised social behaviour
Source: PLoS Comput Biol. 2021 Oct 20;17(10):e1009530. doi: 10.1371/journal.pcbi.1009530 (PMC8559952; doi:10.1371/journal.pcbi.1009530)

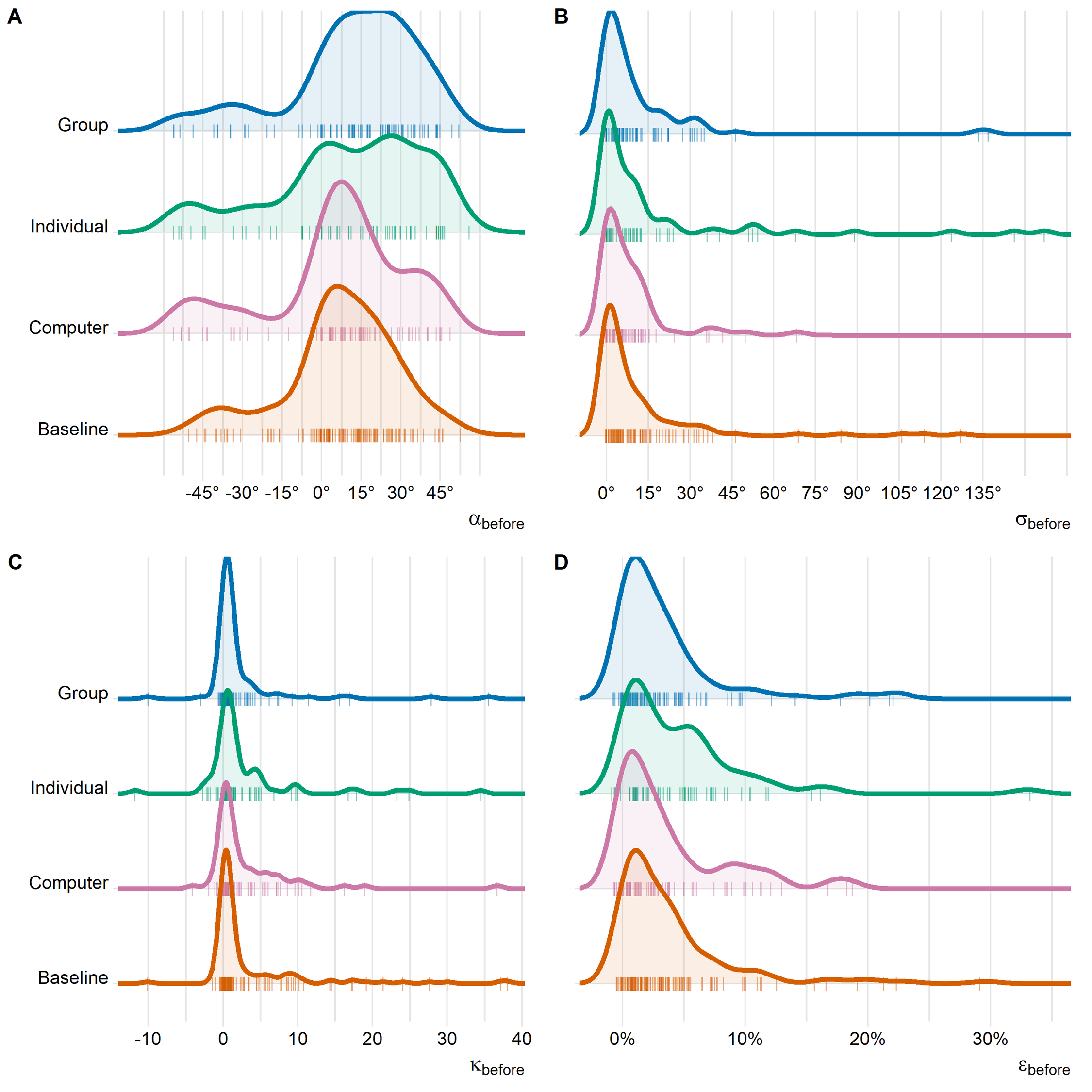

Supplement: S1 Fig — Individual parameters’ distribution before manipulation, by condition. Each vertical line represents a participant, jittered for illustration purposes. Parameter α represents participants’ estimated social attitude; σ estimates participants consistency across choices, κ indicates the penalty points of the default allocation with respect to the alternative allocations; ε indicates the percentage of trials in which there was likely a response mistake by the participant. (TIF) [file pcbi.1009530.s001.tif]
