# Supplementary material for: How conformity can lead to polarised social behaviour
Source: PLoS Comput Biol. 2021 Oct 20;17(10):e1009530. doi: 10.1371/journal.pcbi.1009530 (PMC8559952; doi:10.1371/journal.pcbi.1009530)
Supplement: S1 Methods — (PDF) [file pcbi.1009530.s003.pdf]

S1 Methods. Preregistration amendments

This study was originally preregistered at the Open Science Framework osf.io/th6wp. Although we tried to be as faithful as possible to the original project, we made some changes which we report here:

- Design: the original plan to recruit 100 participants was not reached due to a limited subject pool.
- Hypotheses:
  1. We added the time-dependence hypothesis (participants change attitude even in the absence of another agent).
  2. The norm learning hypothesis does not strictly exclude attitude change in the Individual condition.
  3. New prediction for Preference Learning: increase in consistency.
- Modelling:
  1. Results presented are based on the original 100,000 MCMC iterations, with all parameters well below $\hat{\mathrm{R}} < 1.10$. To achieve the more conservative threshold of $\hat{\mathrm{R}} < 1.05$ for all parameters, we ran 222,500 iterations with thinning = 4, while also excluding one participant from the Baseline condition. Results are statistically equivalent to those presented in the article.
  2. We introduced the bias parameter κ to account for any additional preference for the alternative or the default allocation, *ceteris paribus*. When considering for instance a logistic regression model with choice as the predicted variable, κ can be thought as the intercept of the regression. Previous findings suggest that participants might choose the alternative allocation more often than predicted by the original choice models, given that introducing a fixed option in the choice might bias the expectations of participants on how to respond, regardless of the payoffs (see [1]).
  3. We introduced the error parameter ε, transforming the model into a mixture between the original preference model and an unbiased coin. This parameter accounts for the possibility that, in some trials, participants answer randomly due to attention gaps or motor mistakes (implementation errors [2] that otherwise would be unaccounted for.
  4. We changed the operationalisation of the dependent variable (Section Manipulation Phase).
- Analyses:
  1. We could not test for participants' numeracy as originally planned due to a problem with the software that inflated the number of mistakes by participants. The mistake was due to the program recognising input from the numeric pad as different symbols than digits, thus counting correct inputs as mistakes. We decided to drop this measure since our recruitment schedule prevented us to fix this problem in time for the data collection.

- Names:
  1. Epistemic Uncertainty → Preference Learning
  2. Preference Temperature → Stable Attitude
  3. Preference Uncertainty → Variable Attitude

References

1. Zizzo DJ, Fleming P. Can experimental measures of sensitivity to social pressure predict public good contribution? Economics Letters. 2011;111(3):239–242. doi:10.1016/j.econlet.2011.02.021.
2. Loomes G, Moffatt PG, Sugden R. A microeconometric test of alternative stochastic theories of risky choice. Journal of risk and Uncertainty. 2002;24(2):103–130.
