# Supplementary material for: How conformity can lead to polarised social behaviour
Source: PLoS Comput Biol. 2021 Oct 20;17(10):e1009530. doi: 10.1371/journal.pcbi.1009530 (PMC8559952; doi:10.1371/journal.pcbi.1009530)
Supplement: S1 Table — (PDF) [file pcbi.1009530.s004.pdf]

Threshold	Statistical test							Number of observations				
	Wilcoxon signed-rank W/n_{obs}			Kruskal-Wallis χ^2	Dunn's post-hoc z							
	Computer > 0	Individual > 0	Group > 0		Group ≠ Baseline	Individual ≠ Baseline	Group ≠ Computer	Group	Individual	Computer	Baseline	**Total**
100%	*** 18.01	*** 22.08	*** 32.46	*** 41.98	*** 5.86	*** 4.68	1.76	97	66	73	132	**368**
40%	*** 17.86	*** 19.54	*** 30.57	*** 38.85	*** 5.66	*** 4.40	1.66	93	56	71	123	**343**
35%	*** 15.49	*** 18.91	*** 30.35	*** 35.79	*** 5.52	*** 4.18	1.94	92	55	67	117	**331**
30%	** 12.63	*** 18.28	*** 29.78	*** 34.5	*** 5.46	*** 4.05	* 2.31	91	54	63	116	**324**
25%	* 10.37	*** 16.58	*** 27.52	*** 31.72	*** 5.20	** 3.90	* 2.48	86	50	60	109	**305**
20%	* 9.25	*** 16.58	*** 29.04	*** 38.54	*** 5.81	*** 4.11	** 3.02	82	50	57	104	**293**
15%	* 8.54	*** 15.59	*** 27.04	*** 33.42	*** 5.37	** 3.88	** 2.92	78	46	54	93	**271**
10%	6.85	*** 14.61	*** 25.83	*** 34.76	*** 5.39	** 3.99	** 3.15	72	41	47	85	**245**
