# Supplementary material for: How conformity can lead to polarised social behaviour
Source: PLoS Comput Biol. 2021 Oct 20;17(10):e1009530. doi: 10.1371/journal.pcbi.1009530 (PMC8559952; doi:10.1371/journal.pcbi.1009530)
Supplement: S1 Analyses — (PDF) [file pcbi.1009530.s005.pdf]

S1 Analyses. Model identification

Here we test whether Stable Attitude and Variable attitude models can be identified. Since we were unable to compute a confusion matrix given the high computational resources required to simulate such complex hierarchical models multiple times, we relied on our DIC metric for a single set of simulated data. We first generated data based on both the Stable Attitude and Variable Attitude models, using the same design structure of the experiment and with the same number of participants in each condition. We then fitted the resulting simulated data to both models. As expected, data generated from the Variable Attitude model was fitted best by the Variable Attitude model (DIC = 34128.15) than by the Stable Attitude model (DIC = 37441.68), and data generated from the Stable Attitude model was fitted best by the Stable Attitude model (DIC = 36015.08) than the Variable Attitude model (DIC = 37758.68).
