# Supplementary material for: How conformity can lead to polarised social behaviour
Source: PLoS Comput Biol. 2021 Oct 20;17(10):e1009530. doi: 10.1371/journal.pcbi.1009530 (PMC8559952; doi:10.1371/journal.pcbi.1009530)
Supplement: S2 Analyses — (PDF) [file pcbi.1009530.s006.pdf]

S2 Analyses. Attitude Convergence δ_{diff} Parameter Recovery.

We test whether the winning model Variable Attitude is able to correctly recover simulated data about our main variable of interest, attitude convergence. To this end, we simulate data based on the estimated hierarchical population parameters and generate data for the same number of participants in each experimental condition. We repeat model fitting using the same procedure for real data, and compute the differences between original simulated data and the values recovered by the model. Results reveal a decent recovery of δ_{diff}, with a correlation between simulated and recovered values of $r = .85$ ($[.82, .88]$, Pearson's product-moment correlation, $p < .001$; Fig Figure), a root mean squared error of 5.5° and a root median squared error of 2.4°.

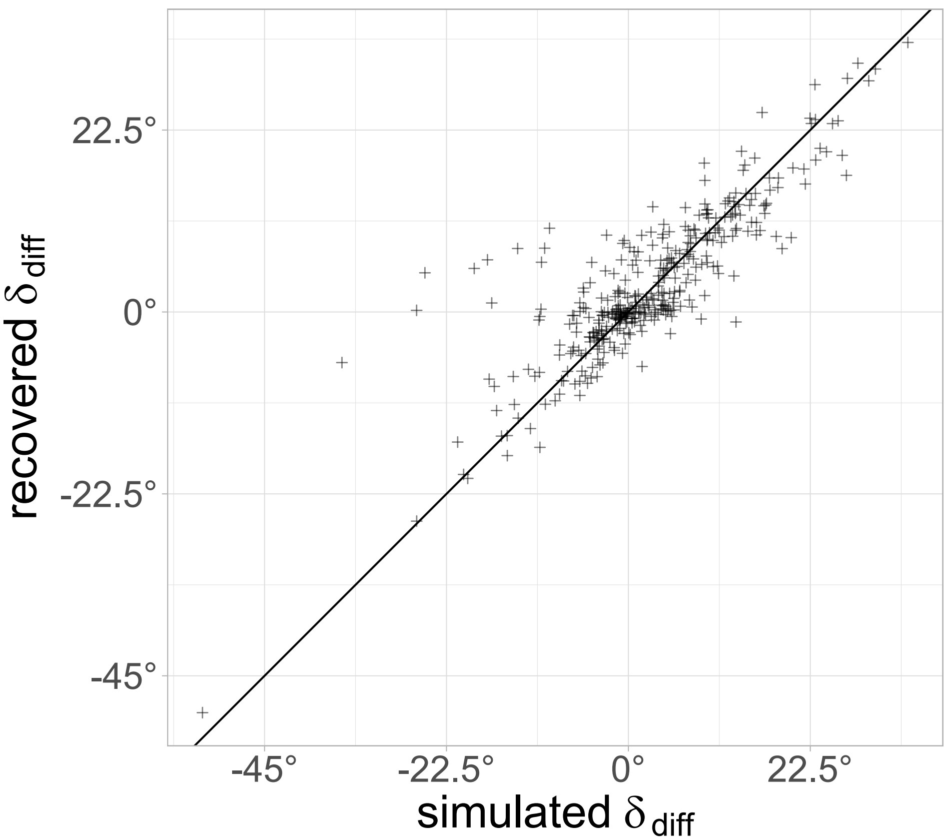

S2 Figure. Parameter recovery for simulated δ_{diff} values. On the x axis are the generated data, on the y axes, the values recovered by the model.
