# Supplementary material for: How conformity can lead to polarised social behaviour
Source: PLoS Comput Biol. 2021 Oct 20;17(10):e1009530. doi: 10.1371/journal.pcbi.1009530 (PMC8559952; doi:10.1371/journal.pcbi.1009530)
Supplement: S3 Analyses — (PDF) [file pcbi.1009530.s007.pdf]

S3 Analyses. Balance Between Conditions

We test whether experimental conditions are balanced in terms of gender, age, and starting social attitude. Whereas we do not find any differences in terms of gender (Chi squared test, $\chi(3) = 1.37$, $p = .713$), we do find that participants in the Individual condition are younger than participants in Baseline (Dunn's post-hoc pairwise comparisons: $W = 2.91$, $p = .015$, $\mathrm{BF}_{10} =$) and Group ($W = 2.80$, $p = .015$, $\mathrm{BF}_{10} =$) conditions. Whereas this differences are statistically significant, in practice the age gap is of around 5 months (Individual: $M = 21$ years, 9 months; Baseline: $M = 22$ years, 3 months; Group: $M = 22$ years, 4 months). We also compare average starting attitude α_{before} for prosocial and antisocial participants by means of a linear regression model with α_1 ranks as predicted variable and condition and interaction between categorisation and condition. We defined pairwise contrasts within each category (i.e., control prosocial participants versus group prosocial participants). All contrasts are not statistically significant (all $p > .163$).
