# Supplementary material for: How conformity can lead to polarised social behaviour
Source: PLoS Comput Biol. 2021 Oct 20;17(10):e1009530. doi: 10.1371/journal.pcbi.1009530 (PMC8559952; doi:10.1371/journal.pcbi.1009530)
Supplement: S4 Analyses — (PDF) [file pcbi.1009530.s008.pdf]

S4 Analyses. Attitude Distance From the Agent Before Prediction

Given that attitude convergence δ_{diff} depends on the initial attitude distance from the agent, we test whether there are any differences in distance across conditions by means of a Kruskall-Wallis rank-sum test (Figure Figure). The test is significant ($\chi^2(3) = 11.52$, $p = .009$, $\varepsilon^2 = .03[.01, .08]$), and post-hoc Dunn's tests reveal that participants in the Group and Individual conditions are on average significantly closer to their agents than participants in the Baseline condition (Group: $p = .015$, $r = .19[.08, .32]$, $\text{BF}_{10} = 4.34$; Individual: $p = .015$, $r = .20[.05, .33]$, $\text{BF}_{10} = 4.27$), whereas there is no difference across other comparisons ($p > .05$). If participants in the Group and in the Individual conditions had an average initial attitude closer to the agent than participants in the Baseline condition, then their maximum possible attitude convergence must have been smaller than that of Baseline participants. Since participants' attitudes in the Individual and Group conditions converge *more* than those in the Baseline condition, this evidence–if anything–supports the idea that differences in attitude convergence across conditions cannot be explained by differing initial distances.

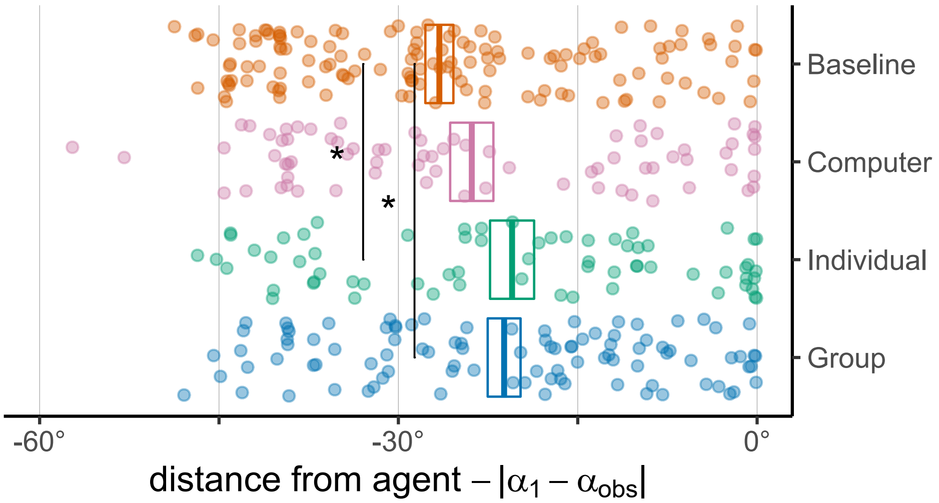

S3 Figure. Distance from observed agent, by condition. Crossbar plot of participants' attitude distance from the agent's before the manipulation phase. The graph includes all participants. Error bars indicate t-adjusted, 95% Gaussian confidence intervals.
