# Supplementary material for: How conformity can lead to polarised social behaviour
Source: PLoS Comput Biol. 2021 Oct 20;17(10):e1009530. doi: 10.1371/journal.pcbi.1009530 (PMC8559952; doi:10.1371/journal.pcbi.1009530)
Supplement: S5 Analyses — (PDF) [file pcbi.1009530.s009.pdf]

S5 Analyses. Time Dependence Analyses

Here we provide quantitative tests for the measures capturing change in the proportion of alternative choices and changes in α. For the former, we consider prosocial trials for participants categorised as prosocial, and antisocial trials for participants categorised as antisocial. We run a mixed-effects logistic regression on these trials, with participant as a random effect, alternative choice (dummy: 1 = alternative chosen) as the predicted variable and, as a predictor variable, a dummy indicating whether the trial was before the manipulation, or if after the manipulation in what given condition (e.g., after the manipulation in the Group condition). According to this model, compared to trials before the manipulation, the proportion of alternatives chosen decreased in the Baseline condition ($\beta = -.242$, Standard Error $S.E. = .040$, $p < .001$), did not significantly change in the Computer condition ($\beta = .073$, $S.E. = .054$, $p = .178$), and increased in the Individual ($\beta = .301$, $S.E. = .056$, $p < .001$) and in the Group ($\beta = .363$, $S.E. = .046$, $p < .001$) conditions.

We additionally test whether participants become more or less polarised after manipulation ($H_1 : \delta_\alpha \neq 0$) Due to model misspecification (i.e., non-normality of data, Shapiro-Wilk test, all $p < 0.039$), we apply non-parametric tests for the analyses. Participants' social attitude became more polarised in all conditions except Baseline (two-tailed Wilcoxon signed-rank test; Baseline: $\log(V) = 8.39$, $p = .990$, $\delta_\alpha = 0°[-1°, 1°]$, $r = .00[-.16, .17]$, $\text{BF}_{01} = 10.19$; Computer: $\log(V) = 7.60$, $p = .001$, $\delta_\alpha = 4°[2°, 6°]$, $r = .38[.18, .59]$, $\text{BF}_{10} = 104.59$; Individual: $\log(V) = 7.46$, $p < .001$, $\delta_\alpha = 7°[3°, 10°]$, $r = .50[.31, .68]$, $\text{BF}_{10} = 826.95$; Group: $\log(V) = 8.24$, $p < .001$, $\delta_\alpha = 5°[3°, 7°]$, $r = .52[.37, .68]$, $\text{BF}_{10} > 10000$).
