# Supplementary material for: How conformity can lead to polarised social behaviour
Source: PLoS Comput Biol. 2021 Oct 20;17(10):e1009530. doi: 10.1371/journal.pcbi.1009530 (PMC8559952; doi:10.1371/journal.pcbi.1009530)
Supplement: S6 Analyses — (PDF) [file pcbi.1009530.s010.pdf]

S6 Analyses. Attitude Polarisation

Here we report the main results of the experiment using attitude polarisation δ_α in place of attitude convergence δ_{diff}. These tests show that the results are robust to the specification of the dependent variable.

Full sample, $H_1 : \delta_\alpha > 0$**.** Due to model misspecification (i.e., non-normality of data, Shapiro-Wilk test, all $p < 0.039$; inequality of variances, Fligner-Killeen test, $p < 0.001$), we apply non-parametric tests for the analyses. Participants' social attitude became more polarised in all conditions except Baseline (one-tailed Wilcoxon signed-rank test; Baseline: $\log(V) = 8.39$, $p = .495$, $\delta_\alpha = 0°[-1°, 1°]$, $r = .00[-.16, .17]$, $\text{BF}_{0+} = 12.40$; Computer: $\log(V) = 7.60$, $p < .001$, $\delta_\alpha = 4°[2°, 6°]$, $r = .38[.18, .59]$, $\text{BF}_{+0} = 209.14$; Individual: $\log(V) = 7.46$, $p < .001$, $\delta_\alpha = 7°[3°, 10°]$, $r = .50[.31, .68]$, $\text{BF}_{+0} = 1653.85$; Group: $\log(V) = 8.24$, $p < .001$, $\delta_\alpha = 5°[3°, 7°]$, $r = .52[.37, .68]$, $\text{BF}_{+0} > 10000$).

Attitude polarisation also differs by condition (Kruskal-Wallis rank-sum test, $\chi^2(3) = 45.52$, $p < .001$, $\varepsilon^2 = .10[.06, .17]$). Baseline differs from all other conditions (Computer: $W = 3.19$, $p = .003$, $r = -.23[-.37, -.09]$, $\text{BF}_{10} = 8.97$; Individual: $W = 4.22$, $p < .001$, $r = -.29[-.43, -.13]$, $\text{BF}_{10} = 107.69$; Group: $W = 5.57$, $p < .001$, $r = -.37[-.49, -.23]$, $\text{BF}_{10} = 4882.74$). Whereas no other difference is significant, there is substantial evidence ($\text{BF}_{01} = 5.78$) for no difference between Group and Individual condition.

Participants with compliance index $<$ **25%,** $H_1 : \delta_\alpha > 0$**.** Due to model misspecification (i.e., non-normality of data, inequality of variances), we apply non-parametric tests for the analyses. For participants below threshold, average attitude polarisation is significantly different from zero in the Individual and Group conditions but not in the Baseline condition, whereas there is not enough evidence for either hypothesis in the Computer condition (one-tailed Wilcoxon signed-rank test; Baseline: $\log(V) = 8.01$, $p = .493$, $\delta_\alpha = 0°[-1°, 1°]$, $r = .00[-.19, .20]$, $\text{BF}_{01} = 9.21$; Computer: $\log(V) = 7.05$, $p = .056$, $\delta_\alpha = 2°[0°, 4°]$, $r = .22[-.02, .47]$, $\text{BF}_{10} = 1.59$; Individual: $\log(V) = 6.93$, $p < .001$, $\delta_\alpha = 5°[2°, 8°]$, $r = .52[.31, .74]$, $\text{BF}_{10} = 591.57$; Group: $\log(V) = 7.97$, $p < .001$, $\delta_\alpha = 5°[3°, 7°]$, $r = .48[.30, .65]$, $\text{BF}_{10} = 2302.02$).

Attitude polarisation also differs by condition (Kruskal-Wallis rank-sum test, $\chi^2(3) = 28.87$, $p < .001$, $\varepsilon^2 = .09[.05, .18]$). Baseline differs from Group and Individual conditions (Individual: $W = 3.75$, $p < .001$, $r = -.17[-.36, -.02]$, $\text{BF}_{10} = 29.10$; Group: $W = 4.92$, $p < .001$, $r = -.34[-.47, -.19]$, $\text{BF}_{10} = 248.05$). Group condition is also statistically different from the Computer condition, although Bayes Factor suggests evidence is anecdotal ($W = 2.53$, $p = .023$, $r = .21[.06, .37]$, $\text{BF}_{10} = 1.16$). Whereas no other difference is significant, there is substantial evidence for no difference between Group and Individual condition ($\text{BF}_{01} = 5.17$).

Robust regression. In support of the compliance hypothesis, we find that the weights for the main effect of Group and Individual conditions are significant, whereas that of the Computer condition is not (Baseline: $\beta = .14[-1.18, 1.46]$, $t = .211$, $p = .833$; Computer: $\beta = -.41[-2.27, 1.44]$, $t = -.438$, $p = .662$; Individual: $\beta = 3.73[1.70, 5.75]$, $t = 3.60$, $p < .001$; Group: $\beta = 4.47[2.95, 5.99]$, $t = 5.75$, $p < .001$). Furthermore, the interaction term with compliance is only significant in the Computer condition ($\beta = 27.17[15.57, 38.77]$, $t = 4.59$, $p < .001$) and not in the other experimental conditions (all $p > .05$).
