# Supplementary material for: How conformity can lead to polarised social behaviour
Source: PLoS Comput Biol. 2021 Oct 20;17(10):e1009530. doi: 10.1371/journal.pcbi.1009530 (PMC8559952; doi:10.1371/journal.pcbi.1009530)
Supplement: S7 Analyses — (PDF) [file pcbi.1009530.s011.pdf]

# S7 Analyses. Attitude Convergence and Consistency Increase

As an exploratory question, we asked whether participants would become more consistent the more they conform. We tested whether attitude convergence and consistency increase were positively correlated by means of a directional Spearman's rank correlation. Correlation was not significant in any condition after correcting for multiple comparisons (all $p > .998$).
