# Supplementary material for: How conformity can lead to polarised social behaviour
Source: PLoS Comput Biol. 2021 Oct 20;17(10):e1009530. doi: 10.1371/journal.pcbi.1009530 (PMC8559952; doi:10.1371/journal.pcbi.1009530)
Supplement: Attachment — Submitted filename: Panizza_reviewers_response.docx [file pcbi.1009530.s012.docx]

Response to referees

We would like to thank both reviewers for their insightful comments. We have modified the manuscript accordingly and included the suggested analyses. We have also adapted the style of the manuscript to the standards of the journal. Changes and responses to reviewers' comments are listed below, and next to them are the lines and pages where the changes were made:

Reviewer #1

“Are the groups of participants matched in terms of sex, age, baseline social preference and other characteristics? In between-participant design, this issue should be carefully checked.

*We have added a supplementary analysis testing for differences in demographics and initial attitude (lines 868-880, page 22).*

“Does contagion or automatic imitation imply conformity occur regardless of whether the observed agent is human or non-human? How about human-specific automatic imitation reported in psychological literature?

*We address this interesting point in the discussion (ll. 657-664, p. 17): “Whereas the experimental design focused on some of the most prominent hypotheses in the literature, it is also possible that further mechanisms may guide participants’ social conformity. Social mimicry for instance suggests that participants change attitude in order to increase ties within a group [14,75,76]. The need to belong [77] could represent an alternative explanation to norm compliance, although it is not able to explain why prosocial and antisocial participants display different appropriateness beliefs. It is also by all means possible that several mechanisms contribute together to the present results; our main conclusion is that they probably operate on a social level.”*

Reviewer #2

“I suggest a stronger focus on the modelling approach, with modelling methods (Supp. Mat. A7) and modelling results (Supp. Mat. B1) incorporated in the main text.

*We have extensively modified the manuscript to include the modelling analyses and results in the main text.(ll.351-422, p. 10; ll. 424-451, p. 12; ll. 669-724, pp. 17-18)*

“[B]ecause a lot of analyses apply to attitude alignment (d_diff), which is a model parameter, the other would need to provide a parameter recovery exercise (see e.g. Wilson and Collins, eLife 2019). A model identification would also be needed to support the model comparison results.

*We performed parameter recovery for δ*_diff_ *for the winning model (full Variable Attitude) and reported the results as a supplementary analysis (ll. 858-867, p. 22). Given the sheer size of the hierarchical models and the limited computational resources available, we did not perform a full model identification. However, we simulated data for a full Stable Attitude and a full Variable Attitude models, and estimated their DIC values when fitted with their homologous and their alternative model. Results are also reported as supplementary results (ll. 846-857, pp. 21-22).*

“Some effects size/direction are missing (e.g. paragraph about attitude convergence differences between conditions p.9)

*We have now added all the effect sizes for pairwise comparisons between conditions (Wilcoxon’s R; lines 498,499,500, page 13; ll. 518,521,522, p. 14; l. 553, p. 15; ll. 887,888, p. 22; ll. 932,933,934, p. 23).*

“To provide a simple, first overview of the main effect of interest, I suggest that the authors also include a Figure (before the current Figure 2) which simply display the attitude convergence in the different conditions

*We now have included a new Figure (now* ***Figure 3****) representing attitude convergence across conditions (p. 13).*

“Fig 3B : Unless I’m missing something, there seems to be an inconsistency between the significance stars and the CI (and the results reported in the Main text)

*The 95% confidence interval is computed using the* smean.cl.boot *function of R package* Hmisc*. Despite being a nonparametric (not assuming normality) bootstrap method, confidence intervals are still affected by the presence of multiple outliers, hence the discrepancy between the statistical test and the confidence interval including the 0 difference. A more appropriate representation of confidence intervals would be based on ranks, given that our statistical tests are rank-based, but we could not find any feasible graphical representation.*

“Paragraph head “consistency increase” should be “preference learning” for clarity/consistency

*We have changed the name of the paragraph accordingly (l. 282, p. 8).*
