# Supplementary material for: How conformity can lead to polarised social behaviour
Source: PLoS Comput Biol. 2021 Oct 20;17(10):e1009530. doi: 10.1371/journal.pcbi.1009530 (PMC8559952; doi:10.1371/journal.pcbi.1009530)
Supplement: Attachment — Submitted filename: Panizza_reviewers_response.docx [file pcbi.1009530.s013.docx]

Response to referees

*Minor issue: I am surprised about the reported effect-size confidence intervals and how they relate to p-values e.g. lines 521-522: “Baseline and Group conditions are still significantly different (z = 5:20, p < :001, 520 r = -.37[-.51; .24], BF10 = 2117:44), and so are Baseline and Individual conditions (z = 3:90, p = :001, r = -:31[-:45; :15], BF10 = 54:87).” Aren’t some “minus” signs missing from the second terms in the brackets (to exclude 0 from the CI and reach high significance levels of P<0.001)? Also would better correspond to the reported mean the center of the CI. Please check throughout the manuscript.*

We thank reviewer #2 for spotting these typographical errors. We have corrected the sign of the upper boundaries of the two confidence intervals. Furthermore, we have checked the other confidence intervals in the manuscript and spotted another mistake at line 952 (supplementary information), where lower and upper boundaries were swapped. We have also corrected this mistake.

We thank again the invaluable work of both reviewers in improving this manuscript to make it suitable for publication.

Best regards,

Folco Panizza
